# Fabrication of Tapered 3D Microstructure Arrays Using Dual-Exposure Lithography (DEL)

**DOI:** 10.3390/mi11100903

**Published:** 2020-09-29

**Authors:** Venkatakrishnan Rengarajan, Junnan Geng, Yu Huang

**Affiliations:** Department of Biological Engineering, Utah State University, Logan, UT 84322, USA; venkatakrishnan.rengarajan@gmail.com (V.R.); junnan.geng@aggiemail.usu.edu (J.G.)

**Keywords:** interference lithography, microarray structures, Cassie–Baxter wetting, superhydrophobic, optical diffraction, grating, cell patterning, protein patterning

## Abstract

Three-dimensional (3D) microstructure arrays (MSAs) have been widely used in material science and biomedical applications by providing superhydrophobic surfaces, cell-interactive topography, and optical diffraction. These properties are tunable through the engineering of microstructure shapes, dimensions, tapering, and aspect ratios. However, the current fabrication methods are often too complex, expensive, or low-throughput. Here, we present a cost-effective approach to fabricating tapered 3D MSAs using dual-exposure lithography (DEL) and soft lithography. DEL used a strip-patterned film mask to expose the SU-8 photoresist twice. The mask was re-oriented between exposures (90° or 45°), forming an array of dual-exposed areas. The intensity distribution from both exposures overlapped and created an array of 3D overcut micro-pockets in the unexposed regions. These micro-pockets were replicated to DEL-MSAs in polydimethylsiloxane (PDMS). The shape and dimension of DEL-MSAs were tuned by varying the DEL parameters (e.g., exposure energy, inter-exposure wait time, and the photomask re-orientation angle). Further, we characterized various properties of our DEL-MSAs and studied the impact of their shape and dimension. All DEL-MSAs showed optical diffraction capability and increased hydrophobicity compared to plain PDMS surface. The hydrophobicity and diffraction angles were tunable based on the MSA shape and aspect ratio. Among the five MSAs fabricated, the two tallest DEL-MSAs demonstrated superhydrophobicity (contact angles >150°). Further, these tallest structures also demonstrated patterning proteins (with ~6–7 μm resolution), and mammalian cells, through microcontact printing and direct culturing, respectively. Our DEL method is simple, scalable, and cost-effective to fabricate structure-tunable microstructures for anti-wetting, optical-, and bio-applications.

## 1. Introduction

A three-dimensional microstructure array (3D-MSA) comprises a large number of micro-structured shapes (e.g., cylindrical [1], cone [2], hemispherical [3], and trapezoidal [4]), arranged in a defined pattern [5]. MSAs, with these micron-sized (~1–15 microns) structural units, bestow unique tunable material properties, such as wettability, optical characteristics, and cell adhesion [6,7,8]. These MSAs have been widely employed in studies like anti-wetting surfaces [9], optical waveguide fabrication [10], cell mechanics studies [11], stem cell engineering [12], and other scientific material applications [2,13]. MSA applications also include structural support in the fabrication of microelectrode array for brain interface studies [14,15], microprobes for neural depth recordings [16,17], microneedles for drug delivery [18], and other micro-optic studies [19,20].

The conventional micromachining technologies (e.g., bulk micromachining [21], surface micromachining [22], and UV LIGA [10]) to fabricate MSAs are inadequate to achieve the desired size or 3D shapes of the microstructures. Besides, commonly used advanced lithography methods (e.g., deep reactive ion etching [23], thermal reflow [24], inclined UV lithography [25], and focused ion beam [6,26]) to achieve these 3D shapes also suffer from limitations as they are equipment-/environment-demanding, expensive, and low-throughput. Recent additive manufacturing techniques (e.g., Inkjet printing [27,28] and direct laser writing [29]) for manufacturing 3D MSAs also face challenges such as nonuniformity, nozzle clogging (that affects the reproducibility of the prints), material choice limitation, complex operation, and low surface finish [28,30,31].

Alternative methods, tweaking the conventional lithography, have been explored [32,33,34] to target the complex 3D-shapes and aspect ratios of MSAs primarily. Although these methods have demonstrated the advantage of thick photoresists’ (e.g., SU-8) 3D-sculptability, they suffer from a series of limitations. For instance, dual diffusers were used to fabricate cone-shaped microstructures but needed an additional setup to control the diffusion angle, MSAs’ dimension, and aspect ratio [4,34]. Backside exposure was employed to achieve various aspect ratios of MSA but was incompatible with commonly used silicon and opaque materials [18,35]. Furthermore, most of these alternative methods cannot be extended to soft lithography for replication as the final features were directly realized on the SU-8 substrate.

**Figure 1:**
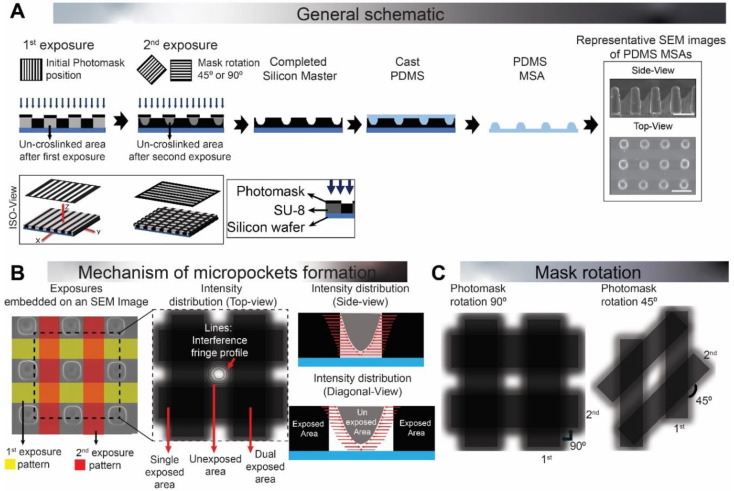
Fabrication of DEL-MSAs (dual-exposure lithography-microstructure arrays). (**A**) General schematic of microstructure array (MSA) fabrication through dual-exposure lithography (DEL). The SU-8 photoresist was exposed twice using a strip-patterned film mask. The initial position of the film mask was at 0° and re-oriented to 45° or 90° (in the xy-plane, along the z-axis) between exposures. The crosslinking energy of two exposures overlapped with each other, creating tapered overcut 3D micro-pockets. Soft lithography was used to mold DEL-MSAs in polydimethylsiloxane (PDMS). (**B**) Mechanism of micro-pocket formation. Left: Patterns of two exposures overlaid on an SEM image, along with an illustration of the intensity distribution from exposed areas to unexposed ones (on x-y axis). Black lines in the unexposed area illustrate the interference fringe pattern. Right: A side view and a diagonal view of the intensity distribution profile (on the z-axis). Red lines indicate the anisotropic intensity distribution in the unexposed areas. The aspect ratio of the micro-pockets was tuned by tuning the fabrication parameters (exposure energy, angle of rotation, and wait time between exposures). (**C**) Top-view schematic of mask rotation effect on the intensity distribution, forming different borders of microstructure. Black arrows denote the rotation angle between first and second exposure. Scale bar: (**A**) 100 μm; (**B**) 10 μm.

In this article, to overcome the limitations described above, we introduce a new method of fabricating MSAs based on dual-exposure photolithography and soft lithography. This method is simple, cost-effective, and tunable in achieving various shapes and aspect ratios of MSA. Negative photoresist (SU-8) was exposed twice with a stripe-patterned film photomask by re-orienting the mask between each exposure, creating an array of dual-exposed areas (Figure 1A). This dual-exposed SU-8 forms an array of 3D micro-pockets that was replicated as DEL-MSAs using soft lithography. The micro-pocket dimensions are tunable through fabrication parameters such as exposure energy, photomask’s rotation angle, and wait time between the two exposures. The surface property of these structural arrays was assessed to reveal its surface hydrophobicity. Additionally, we demonstrated the engineering and biological application of the MSA through optical diffraction, cell patterning, and microcontact printing studies.

## 2. Materials and Methods

### 2.1. Photomask Design

The photomask, which comprised parallel stripes of 50 µm width and 50 µm spacing (Figure 1A), was designed using AutoCAD (Autodesk, V23) and printed through Fine-Line imaging services (Fine-Line imaging, Colorado Springs, CO., USA) in a 20 µm resolution.

### 2.2. Fabrication of SU-8 Master

Fabrication parameters were optimized around the manufacturer’s protocol as base reference (Kayaku Advanced Materials, Westborough, MA, USA) [36]. A 250 µm thick SU-8 2100 (Kayaku Advanced Materials, Westborough, MA, USA) was coated on a silicon wafer (UniversityWafer Inc., Boston, MA, USA) followed by prebaking (5 min at 65 °C and 60 min at 95 °C). The prebaked wafer was cooled to room temperature and was exposed twice under UV radiation using a stripe-patterned photomask. An LED mask aligner (Midas Systems, Yuseong-gu, Daejeon, South Korea) under vacuum contact mode was used to avoid an undesired effect by Fresnel diffraction [37]. The parameter variation implemented to achieve different samples is shown in Table 1. The exposed wafer was then post-baked (1 min @ 65 °C and 25 min @ 95 °C) and developed (25 min, RT) (1-Methoxy-2-Propanol Acetate, Kayaku Advanced Materials, Westborough, MA, USA). Finally, the resist was IPA-cleaned, air-dried, and silanized using 97% 1H,1H,2H,2H-Perfluorooctyltrichlorosilane (Alfa Aesar, Haverhill, MA, USA).

### 2.3. PDMS-MSA Fabrication

A 10:1 (base: initiator) polydimethylsiloxane (PDMS; Sylgard 184, Dow-Corning, Midland, MI, USA) was poured onto the SU-8 masters and degassed thoroughly. After curing for two hours at 120 °C, the PDMS-MSAs were peeled and cured further at 50 °C. Afterward, PDMS-MSAs were cleaned with ethanol and DI water (10 min each) and dried using N_2_.

### 2.4. MSA Structural Analysis

SEM images of the MSAs were taken with FEI Quanta FEG 650 Scanning Electron Microscope (SEM) (ThermoFischer, Waltham, MA, USA). From these images, the height and width of the individual microstructures were analyzed using ImageJ software (V1.53a).

### 2.5. Surface Hydrophobicity Analysis

Surface hydrophobicity analysis was performed as previously described [9]. Briefly, a 2 µL volume droplet was placed on the MSA surface. Droplet profiles, taken using a manual contact angle Model 100SB goniometer (Sindatek, New Taipei, Taiwan) microscope, was used to measure the liquid-solid–air interface contact angle using the ImageJ. Measured contact angles were plotted as a function of the microstructures’ height. Further, the roll-off angle and hysteresis were calculated, as reported previously [1,9].

### 2.6. Angle-Resolved Far-Field Diffraction Analysis

The PDMS DEL-MSA gratings were aligned normal to the incident green laser beam (λ = 570 nm). The diffracted beam was projected on a screen (55 cm from the sample), and the diffraction patterns were captured using a DSLR (Digital Single Lens Reflector) camera (Nikon, Tokyo, Japan). The thickness of the PDMS was kept constant (2 mm) throughout this study. The diffraction angle, *θ_D_*, was calculated using the formulas:

(1) $$\theta_{D}=Sin^{-1}\left(\frac{a}{b}\right)$$

(2) $$b=\;\sqrt{a^{2}+c^{2}}$$

where *a* was calculated using ImageJ as the distance between 0th and 1st order diffraction, and *c* was 55 cm. In this study, only the first-order diffraction angles were calculated and reported.

#### Intensity Profiling

The intensity profile was analyzed from the diffraction pattern images using ImageJ and was plotted as a function of the diffraction angle. In addition, the angle of diffraction along the brightest intensity path was also measured using ImageJ.

### 2.7. Cell Seeding and Imaging on MSA

#### 2.7.1. Coating of PDMS and Fibroblasts Seeding

PDMS molds were cleaned (ethanol—30 min and DI water—10 min), sterilized (Autoclave, Tuttnauer, Hauppauge, NY, USA), and coated with fibronectin (10 µg/mL, Fn, Sigma, St. Louis, MO, USA) for two hours at room temperature in a sterile environment. Afterward, samples were washed with sterilized DI water (3 × 10 min), dried at room temperature, and stored at 4 °C until further use. The mammalian cells (Mouse fibroblast cell line, L929, ATCC, Manassas, VA, USA) were cultured and maintained as previously described [38]. Cells were passaged at 100% confluency using trypsin-EDTA (Sigma, St. Louis, MO, USA). For each coated DEL-MSA substrate, a cell density of 5 × 10^5^ cells/mL were seeded.

#### 2.7.2. Cell imaging and Analysis

After 24 h from cell seeding, cells were stained with live/dead imaging as previously described [38] and imaged using a confocal imaging system (Carl Zeiss, Oberkochen, Germany). Live cell percentage was measured from Z-stack images as described elsewhere [39]. A 3D projection of cells was generated using ImageJ (3D Plugin, V3.96) for Pillar and Bullet substrates. From these images, the position of cells from the center of the microstructure was measured using ImageJ.

### 2.8. Microcontact Printing

Microcontact printing was performed as previously described [40]. Briefly, PDMS stamps were coated with FITC-conjugated poly-l-lysine (PLL-FITC, 1 mg/mL in DI water, Sigma, St. Louis, MO, USA), and were brought in conformal contact with a cleaned glass coverslip with (20 g) and without extra weight. These patterned fluorescent dots were imaged using an optical microscope (Nikon, Tokyo, Japan), and printed pattern measurements were calculated using ImageJ.

### 2.9. Statistical Analysis

Statistical significance between sample means was determined using Student’s *t*-test, and between groups was determined by One-Way ANOVA followed by Tukey’s posthoc analysis for individual comparisons. Statistical tests were performed either in SPSS (IBM, Armonk, NY, USA) or GraphPad (GraphPad, San Diego, CA, USA).

## 3. Results and Discussion

### 3.1. Size and Shape Tunability of the MSAs

Our DEL method exposed the SU-8 photoresist twice (Figure 1A) using an identical stripe-patterned mask, which was re-oriented before the second exposure (in the xy-plane along the z-axis) to form a grid pattern. The subsequently formed grid pattern consisted of regions with different levels of exposures: more in overlapped crossing areas and comparatively less in the non-overlapped stripes (Figure 1B). Intensity distribution from the interference fields of both exposures (in the overlapped areas) [41,42] resulted in an array of 3D micro-pockets in the unexposed areas (Figure 1A,B).

We controlled this intensity distribution by tuning various fabrication parameters (summarized in Table 1) and achieved five microstructure shapes: Dome, Petal, Bell, Bullet, and Pillar. The Dome microstructure parameters were taken as the base, to which modifications were made to fabricate other microstructures. Namely, photomask orientation was changed from 90° to 45° to achieve Petal microstructures (Figure 1C). To test the impact of exposure energy, we halved both the exposure energies of Dome to fabricate Bell microstructure. Wait time was added between exposures (of Dome) to achieve Bullet microstructure. Finally, the synergistic effect of wait time and less exposure energy was tested in the fabrication of Pillar microstructures. These MSAs were then reverse molded using PDMS soft lithography.

**Figure 2:**
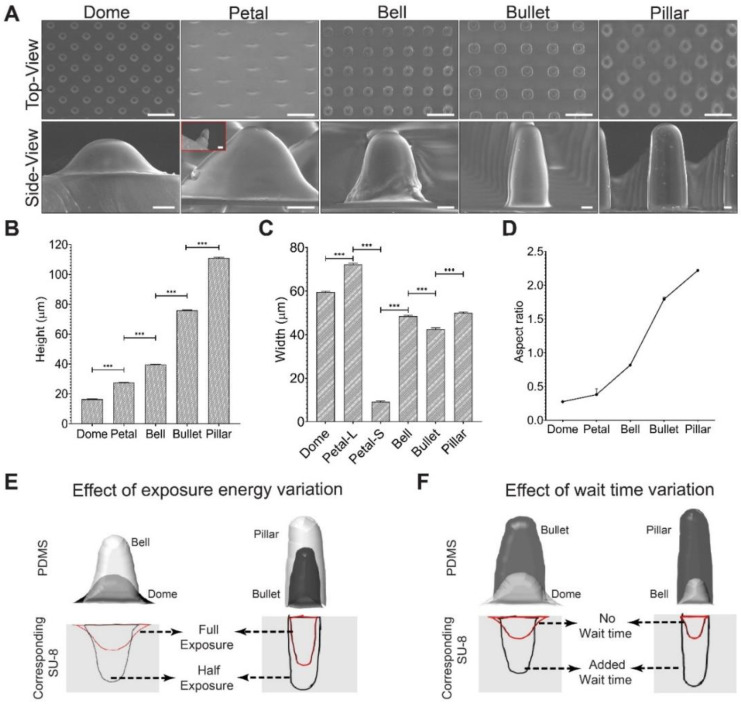
SEM characterization of the PDMS-MSA shape. (**A**) SEM images of the various DEL-MSAs. Petal side-view inset shows the shorter width of the sample viewed from the orthogonal direction, which is much thinner and thus rendering a petal shape. The distribution of (**B**) heights, (**C**) widths and (**D**) the aspect ratios of all fabricated microstructures are shown. Petal is anisotropic in widths. Petal-L and Petal-S denote the longest (72.16 ± 0.612 μm) and the shortest (9.432 ± 0.17 μm) widths, respectively. (**E**) Effect of exposure energy on the microstructure profile. Higher exposure yielded more overcut in SU-8, resulted in shorter and more tapered MSAs. (**F**) Effect of wait time. The addition of wait time leads to much taller and less tapered MSAs. Error Bars: (**B**–**D**) STDEV. Statistical analysis: (**B**,**C**) Student’s *t*-test between groups (*** represents *p* < 0.001). Scale Bar: (**A**) top: 100 μm; bottom: 10 μm.

Exemplary top and side-view SEM images of different PDMS MSAs are shown in Figure 2A, and the corresponding structural attributes are shown in Figure 2B–D. Dome-shaped MSAs, fabricated using base parameters, had an average height of 16.34 µm, and a width of 59.5 µm (measured at the base of the microstructure). The resulting tapered-SU-8 profile (Figure 2E) suggests that the intensity distribution from both the exposures spread in an anisotropic manner (along the z-axis) onto the unexposed areas.

The microstructure shapes were modified (in the xy plane) through changing the photomask rotation angle to 45°, which rendered a Petal-like MSA (Figure 2A). As the name suggests, like a flower petal, this microstructure had a larger width (measured at the base) (72.16 μm; Petal-Large or Petal-L) in the front view and a smaller width (9.432 μm; Petal-Small or Petal-S) in the side view and a height of 27 µm (Figure 2A). This asymmetrical base of the Petal, which contrasts with the Dome (symmetric base), was due to the orientation of interference fringes created during mask re-orientation. Different photomask re-orientation angles result in different shapes of interference lattice [43,44]. In our study, 90° rotation had symmetric interference fringe alignment that rendered cubic lattices, while in 45° rotation, the asymmetric fringe alignment rendered rhombus lattices (Figure 1C). The shorter diagonal of the rhombus lattice promoted shorter width (9 µm), and the longer diagonal promoted larger width (72 µm) (Figure 1C) of the Petal structure.

Further, to test the effect of exposure energy, we reduced both the exposure energies of Dome, which almost doubled the height of the PDMS microstructures (Bell microstructure; height = 39.5 µm) (Figure 2A,B,E). In other words, reducing exposure energy resulted in deeper SU-8 micro-pockets. The depth of the micro-pocket is dependent on the depth of the interference field, which, in turn, is sensitive to the exposure energy [42,45,46,47,48]. Thus, reducing the exposure energy resulted in a weaker interference field (i.e., weaker intensity spread) in the unexposed regions, resulting in deeper SU-8 micro-pockets. This result shows that exposure energy had substantial control over the SU-8 profile and its subsequent PDMS microstructure shapes.

Similarly, adding an interval between the exposures yielded a much taller microstructure (Figure 2A,B,F). When the wait time was introduced between exposures (on top of the mask rotation time) (Dome to Bullet, Bell to Pillar, Figure 2F), the resulting microstructures became much taller with a nearly four times (Bullet; height 75 µm), and three times (Pillar; height 110 µm) increase, indicating much deeper SU-8 micro-pockets. This result is likely caused by the free-radical quenching during photopolymerization, as reported in previous studies [49,50,51,52]. We speculate that through the wait-time (on top of mask rotation time), the free radicals formed during the first exposure could have likely been quenched and reduced the number of free radicals present for further resist polymerization. This, in turn, could have increased the height of the 3D micro-pockets formed. However, the full effect of wait-time will be investigated in future studies.

Interestingly, the wait time showed a more dramatic effect on the microstructures’ dimensions (3–4 times increase) than the reduced exposure dose (two times increase). Decreasing the exposure energy had an inverse effect on the height of the microstructure while adding wait-time between exposures had a direct effect on the height of the microstructure (Figure 2A,B). Furthermore, a combination of lower exposure dose and added wait time resulted in the tallest MSAs (Pillar). Conversely, null wait time and a higher exposure dose yielded the shortest MSAs (Dome). These results show the synergistic effect of reduced exposure dose and wait time on the structural attributes of MSAs, where having both these parameters (reduced exposure or wait time) resulted in the tallest structures compared to the MSAs that used either one of these parameters.

All MSAs, fabricated using the DEL technique, showed significantly tapered 3D micro-pockets (Figure 2A) because of the intensity distribution from the interference field in dual-exposed areas (Figure 1B). Because of this effect, the structures formed on the SU-8 were V-shaped (overcut effect). The same structures cannot be achieved with a checkered patterned photomask (exposing photoresist from top), as it may lead to an undercut effect, with an inverted V-shape structure in the SU-8 [53]. It is also noteworthy that the second exposure polymerized a part of non-exposed areas along the z-direction, starting near the silicon wafer edge. As a result, the heights of the samples were notably lower (16–110 µm) compared to the height of the SU-8 coating (~250 µm).

Nevertheless, we substantially demonstrated that by controlling exposure energy and area of the dual-exposed regions, we could control the aspect ratio and shape of the microstructures. Namely, simple tuning of the photomask rotation (to 45°) rendered a taller and higher aspect ratio microstructure (Petal) for the given exposure parameters (Dome) (Figure 2D). Further, small energy changes led (reducing exposure energy or adding wait time or both) to significant profile change, which resulted in a higher aspect ratio of microstructures.

Collectively, our DEL method demonstrated high versatility and tenability. Using the same photomask, we achieved various shapes of MSAs by merely tuning the exposure energy inter-exposure wait time and orientation of the photomask. Furthermore, unlike other backside-exposure methods that used photoresist as the MSA material [4,32,35], crater-shaped micro-pockets enabled us to use PDMS, which is superior because it is chemically inert, cost-effective, and easily reproducible [54]. Besides, resin thickness can be adjusted to vary microstructure height, as the height of microstructures depends on resin thickness as well. These fabrication parameters can be easily tuned to fabricate different shapes and aspect ratios of micro/nanostructures.

### 3.2. Dimensional Effect of the MSAs on Surface Hydrophobicity

Superhydrophobic properties of natural surfaces, known as the lotus effect, have been extensively mimicked for many industrial and biological applications [55]. MSAs are ideal candidates for such superhydrophobic surfaces because of the densely packed microstructures [1,9]. A direct measure of the MSA’s superhydrophobicity is a high contact angle (>150°) [56] and a low roll-off angle (2–30°) [57], which determine the wettability of the surface [9,58]. Thus, we measured the surface wettability of our MSAs through contact angle and roll-off angle analysis.

**Figure 3:**
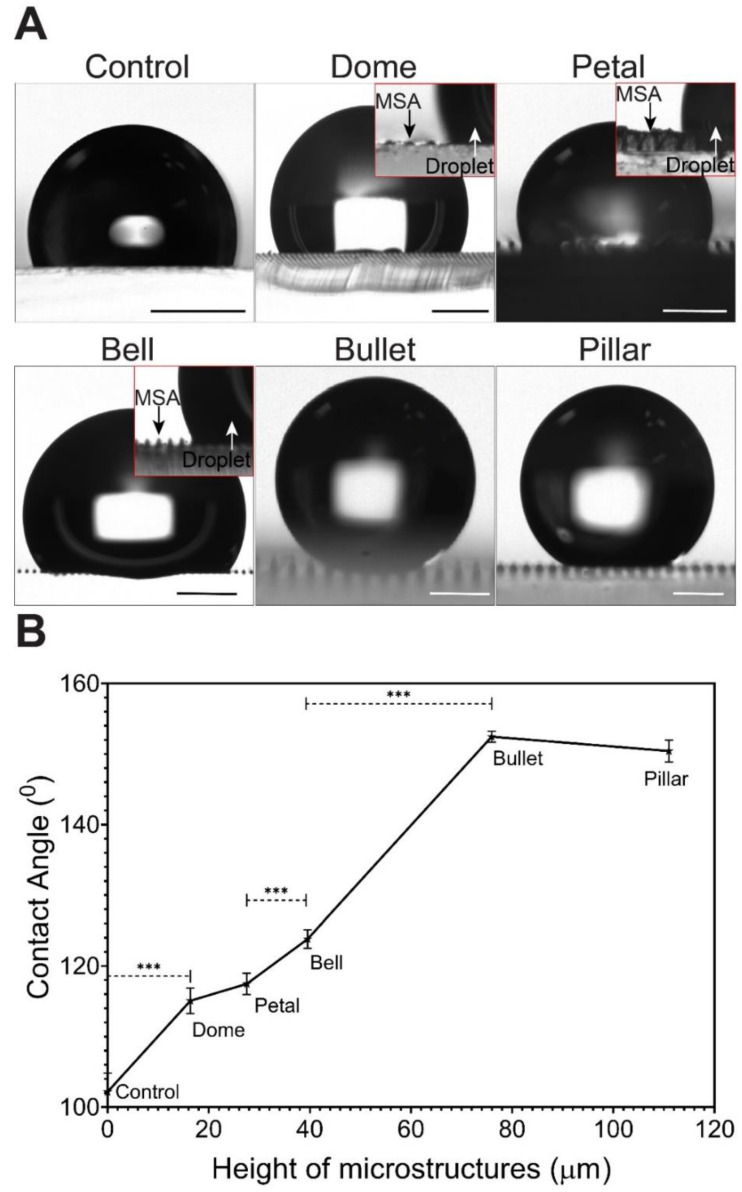
Contact Angle Measurement. (**A**) Microscopic images of the water droplet interaction with the MSAs. Dome, Petal, and Bell showed the Wenzel state (completely wetted surface), while Bullet and Pillar exhibited the Cassie–Baxter state (non-wetted surface), as seen in the lotus effect. Wenzel state of Dome, Petal, and Bell MSAs were shown in the inset images. (**B**) Contact angle values as a function to microstructure height. Bullet and Pillar show superhydrophobic properties (contact angle above ~150°), whereas other samples show higher hydrophobic properties than the control. The contact angle values are linearly proportional to the height (R^2^ = 0.98), with all samples but Pillar. R^2^ = 0.92 if including Pillar. Scale bar: (**A**) 1 mm; Bullet and Pillar: 0.5 mm. Error bars: (**B**) STDEV. Statistical analysis: One-way ANOVA to see the total effect of height on the hydrophobicity (*** *p* < 0.001).

First, we measured the contact angle (CA) of DI water droplets on our MSAs. All our MSAs showed higher hydrophobicity than the control (plain PDMS, CA = 102°) (Figure 3A). While Dome, Petal, and Bell MSAs showed only a slight increase in hydrophobicity (CA = 115°, 117°, and 123°, respectively) than the control, Bullet and Pillar MSAs showed superhydrophobicity, with a much higher contact angle (152° and 150°, respectively). Notably, the contact angle values were directly proportional to the height of the microstructure (Figure 3B).

**Figure 4:**
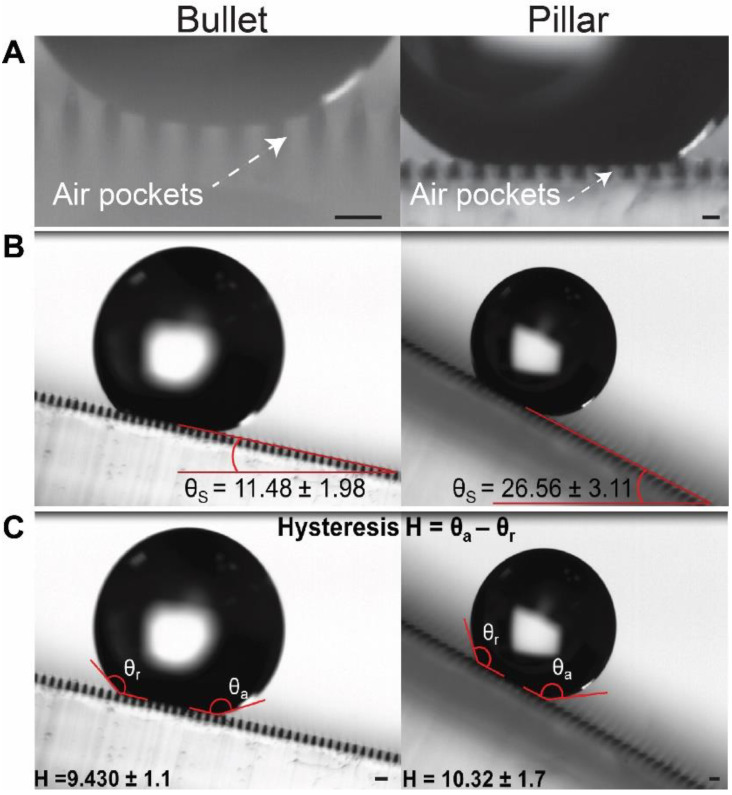
Characterization of the Cassie–Baxter regimen and surface adhesion. (**A**) The air pockets, trapped between the microstructures in the Bullet and the Pillar MSAs, establish that these structures were in Cassie–Baxter wetting condition. Further confirmation of superhydrophobicity was shown through (**B**) the roll-off angle and (**C**) hysteresis analysis. The low roll-off angle and hysteresis define that these two structures formed a superhydrophobic surface. Scale bar: (**A**,**C**) 100 µm.

Next, we determined the wetting state of our MSAs. In our study, two wetting modes are possible based on the contact angle measurement: the wetted Wenzel state and the non-wetted Cassie–Baxter state [1,59]. Dome, Petal, and Bell showed Wenzel state wetting as the droplet recedes into the cavities between the microstructures (Figure 3A: Inset images). On the contrary, Bullet and Pillar MSAs showed Cassie–Baxter wetting, where the droplet was suspended on the rough peaks of the microstructures (Figure 4A). Taken together, the contact angle measurements and the wetting regimen test consistently showed that the Bullet and Pillar MSAs were superhydrophobic.

Additionally, these two MSAs also showed low roll-off angles (Bullet structure (~10°) and the Pillar structures (~26°)), as revealed by the immediate sliding of the droplet after a gradual tilt of the surface (Figure 4B). Furthermore, the hysteresis was also measured to be as low as ~10° for both superhydrophobic surfaces (Figure 4C).

Microscale roughness, according to the lotus effect, is mainly responsible for the level of hydrophobicity of the surface [1,9,59]. With our MSAs, the microscale roughness was directly related to the height of the microstructures, given the same grid distance. This property also influenced the droplet spread on the surface, which inferred the wetting state of the surface: Wenzel or Cassie–Baxter. Here, the shorter microstructures (Dome, Petal, Bell) had relatively lower hydrophobicity and higher surface wetting because the water droplet permeated into the microstructural interstices and increased the contact with the microstructure sidewall surfaces, leading to the Wenzel regimen. In contrast, the taller microstructures (Bullet and Pillar) increased the distance between the water droplet and the surface by trapping air pockets (Figure 4A), which led to no significant impregnation of the water droplet, rendering a Cassie–Baxter regimen. In addition, the low roll-off angles and hysteresis showed that the surface adhesion of the Bullet and Pillar MSAs were low, which is another crucial characteristic of superhydrophobic and self-cleaning surfaces.

Contact angle values are dependent on the microstructure height and the tip radius of the microstructure peak [9], which is the droplet’s primary contact area. A higher tip radius leads to a higher contact area, which lowers the hydrophobicity. Hence, Pillar MSAs, with a higher tip radius (7 µm), showed a lower contact angle, compared to Bullet MSAs (tip radius = 6.5 µm), irrespective of being taller than the Bullet (Figure 3B). The same phenomenon can be extended to explain the higher roll-off angle of the Pillar MSAs compared to the Bullet MSAs. We theoretically ensured this phenomenon by calculating the apparent contact angle from the Cassie–Baxter regimen [9,60] (detailed calculations are in supplementary information). The calculated contact angles for Bullet and Pillar (152° and 148°, respectively) are closer to the observed value, proving the Cassie–Baxter wetting regimen of Bullet and Pillars.

Collectively, we have shown that using the DEL technique, wetting of the MSAs can be tuned to hydrophobic and superhydrophobic surfaces. Our results were obtained in no-coating conditions. Hence, further hydrophobic coatings can be employed to increase DEL-MSAs’ hydrophobicity based on the desired application.

### 3.3. Optical Diffraction Application of the MSA

MSAs are in high demand in optics and photonics, as their optical properties can be tuned through microstructures geometry [10,61,62,63]. PDMS elastomer-based gratings are particularly popular for their advantages (e.g., UV-vis transparent, cost-effective, consistent, and chemically stable), and thus used in many biomedical applications (e.g., thermal detectors [19], glucose monitoring devices [62], cell interaction [20], microfluidics [13], and spectrophotometers [64]). Hence, we tested the ability of our PDMS DEL-MSAs as a transmission-phase grating as it has unique surface properties.

**Figure 5:**
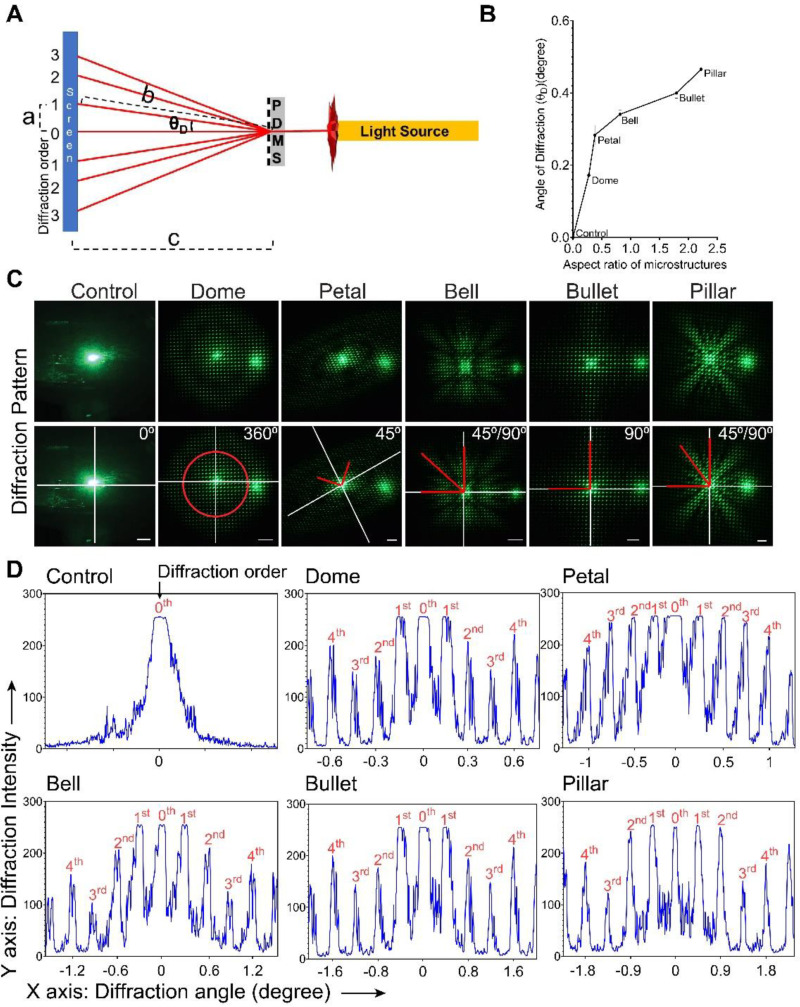
Characterization of optical diffraction of a monochromatic wavelength passing through the MSAs. (**A**) Schematic of the optical arrangement to determine diffraction angle. “a” is the distance between 0th (un-diffracted light) and 1st (diffracted light) order of diffractions. “c” is the distance between the MSA and the screen. “b” was calculated based on a and c values. Using these values, the 1st order angle of diffraction, *θ_D_*, was calculated as the angle between the un-diffracted and first-order diffracted light. (**B**) The angle of diffraction, *θ_D_*, as a function of the MSA aspect ratio. (**C**) Top row: Diffraction pattern of the monochromatic light through the DEL-MSA grating. Note that the brightest intensity observed on the right side in the diffraction images was an artifact that occurred during image capturing and was not considered for any calculations or conclusions. Bottom row: The angle of diffraction along the brightest intensity path was notated: white lines indicate the x- and y-axis passing through the 0th order of diffraction, which was the reference to calculate the diffraction pattern angle (indicated by the red lines). The numbers indicate the brightest light diffraction angle. (**D**) Intensity profile of the diffraction pattern (up to 4th order) as a function of diffraction angle. Error bars: (**B**) STDEV. Scale bar: (**C**) 3 cm.

Angle-resolved far-field diffraction was characterized for all PDMS MSAs via diffraction angle of monochromatic green light (λ = 570 nm) (Figure 5A). The first-order diffraction angle, *θ_D_*, was calculated from Equations (1) and (2) and presented in Figure 5B. The observed first-order diffraction angle was ranging from 0.17° to 0.46°, Dome to Pillar, respectively. This result was theoretically verified based on Bragg’s law [62] (detailed calculation in supplement information). The theoretically calculated value, 0.17°, was in the observed diffraction angle range (0.17°–0.46°). Besides, this result underlines the ability of our DEL technique to tune diffraction angles by engineering different shapes of MSAs.

Furthermore, we measured the angle of diffraction along the path of a brighter intensity (Figure 5C). The control (plain PDMS) surface had no diffraction pattern, whereas MSAs showed a defined diffraction pattern (Figure 5C). A 3D view of these diffraction patterns is represented in Figure S1 for a better view. Specifically, Dome MSAs (least height) showed circular, 360° diffraction patterns, while the Petal MSAs showed 45° diffraction patterns. It is noteworthy that the diffraction angle of Petal was similar to its photomask rotation angle during fabrication. More interestingly, Bell and Pillar MSAs, fabricated using reduced exposures, showed similar diffraction patterns (45° and 90°). Bullet structures, fabricated using similar parameters of Dome structure with added wait time between exposures, showed a clear 90° diffraction pattern. On the whole, these results show that the primary diffraction property of the surface can be tuned through fabrication parameters. Note that the brightest intensity observed on the right side in the diffraction images was an artifact that occurred during image capturing and was not considered for any calculations or conclusions.

In addition, we calculated the intensity profile of diffraction orders (up to 4th order) to determine the light-resolving capacity of our MSAs (Figure 5D). Here, the 0th order of diffraction represents un-diffracted light, and the following diffraction order (1 to *n*) represents diffracted light. In our results, all our MSAs showed almost equal diffraction intensity from 0th to 2nd order. Interestingly, Bell and Pillar’s gratings showed repeating higher intensities in higher diffraction orders (around 10th order) (Figure 5C). These results show that a unique diffraction pattern can be generated using our MSAs that can be employed in various optical studies.

Overall, we have demonstrated an inexpensive and potentially portable optical sensing platform based on our DEL-MSAs, which can be used as transmission gratings for near-infrared waveguided applications. Moreover, the optical properties of our MSAs are easily tunable through the fabrication parameters, which can be used to fabricate microscale gratings with different optical properties. Like previously reported MSAs [19,20,63,65], our MSAs also had a novel angle and dimensions to convert a light signal into a visible diffraction pattern that was discernable to naked eyes. In addition, light diffracted to higher orders was desired in an elastomer-based grating [66,67] because, by selecting or blocking these higher orders, the diffracted light can be better modulated and understood [66,68,69]. Notably, in all DEL-MSAs, higher-order diffractions (up to 4th) had more than 20% diffraction efficiency (ratio of diffracted intensity to the incident light). More interestingly, some of our MSAs showed a repeated higher intensity at a much higher diffraction order (around 10th order of diffraction) (Figure 5C and Figure S1), which can efficiently modulate the intensity of longer wavelength signals [68,69]. Furthermore, our DEL-grating can be extended to stimuli-responsive polymers that are highly reactive to external stimuli (example, pH, temperature, or chemical changes) [62,65]. For instance, external stimuli modify the structural attributes of the microstructures (diameter and distance between the microstructures), which can be accurately reflected in a diffraction pattern [65]. In other words, by extending our DEL-MSAs to stimuli-responsive polymers, minor or invisible changes in the physicochemical parameters (that are invisible to the naked eye) can be converted and visualized through visible and testable diffraction patterns [10,19,20,62,65]. The provided versatile sensing capabilities can pave the way for the use of DEL-gratings as photonic sensors.

### 3.4. MSA as Cell Culturing Substrates

MSAs with topographical and elasticity components are frequently used for cell and tissue engineering to analyze cell adhesion, proliferation, migration, and stem-cell differentiation properties [8,11,12,20]. Specifically, PDMS MSAs are now frequently used to closely mimic mechanical physiology by tuning the substrate stiffness [11]. Hence, we tested the ability of our DEL-MSAs as a substrate for cell culturing and patterning.

**Figure 6:**
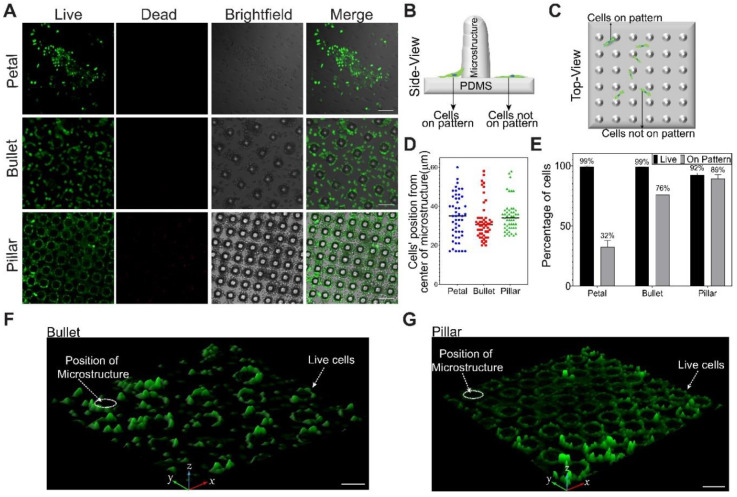
Fibroblast cell patterning on the MSAs. (**A**) Microscopic images revealed a weak, moderate, and strong patterning of fibroblast cells on Fn-coated Petal, Bullet, and Pillar, respectively. Cells showed high viability (virtually no dead cells) on all the array structures. (**B**) Side- and (**C**) top-view schematics illustrated the definition of cells on the pattern and not on the pattern (off-pattern). (**D**) Scatter plot of the cell distance from the center of the microstructure. Scale-0 of the Y-axis represents the center of the microstructure. Bullet and Pillar showed cells mostly arranged close to the microstructure, but Petal showed a random distribution of cells around the microstructures. (**E**) Cell patternability and viability in percentage. (**F**,**G**) 3D projection of live cells on the Bullet and Pillar substrates revealed that the cells attached to the pattern showed upward elongation along the walls of the microstructure. Error bars: (**E**) STDEV. Scale bar: (**A**) 100 μm; (**F**,**G**) 50 μm.

We chose three MSAs as cell substrates for this study: Petal, Bullet, and Pillar, to have three different patterning and microstructures’ area. We first sought to determine the biocompatibility of our substrates by culturing mouse fibroblast cells on them after Fn coating. After 24 h, cells adhered to all the MSA surfaces (Figure 6A).

Further, the cell-patterning effect of MSAs was characterized using two results: cell position around the microstructure (Figure 6B–D) and cells showing preferential adhesion to the pattern (Figure 6E–G). On Petal substrates, cells were mostly distributed on the flat un-patterned area, while on Bullet and Pillar substrates, cells were close to the microstructures (Figure 6A,D,F,G). Furthermore, a higher percentage of cells followed the pattern on the Bullet and the Pillar substrates compared to the Petal substrate (Figure 6E).

Additionally, we determined the cell viability percentage on these substrates through live/dead analysis. Petal and Bullet substrates showed a high cell viability of around 99%, whereas the Pillar substrate showed a slightly lower cell viability of 92% (Figure 6A,E).

Taken together, these results demonstrated the biocompatibility of our substrates for tissue attachment for any cellular studies. Given that the entire area of all the substrates was coated with Fn, there was an evident topographical effect among the substrate on the cell patterning. This result can be attributed to the spatial distribution (arrangement) of the microstructures. An equidistance and closely arranged microstructure pattern (Bullet and Pillar) provided a tight junction for the cells to attach, unlike a non-equidistant and zig-zag pattern (Petal). These results show that our MSA can provide topographical guidance for cell attachment and well-aggregated patterns, which are beneficial for cell and tissue engineering [70,71].

Furthermore, on Pillar MSAs, a higher percentage of cells were on the pattern compared to the Bullet structure. The higher aspect ratio of Pillar MSAs provided higher curvature, maximizing the cytoskeletal tension of the cells [72]. This increase in the cytoskeletal tension, in turn, increased the number of cells adhered to the pattern [72]. Besides, the 3D projection imaging showed cellular elongations upward along the walls of Bullet and Pillar microstructures (Figure 6F,G), which suggests that our DEL-MSAs can be useful to study the cell behaviors in a 3D microenvironment, as well.

Appropriated from our DEL technique, a few minor modifications to the photoresist’s thickness and the photomask’s line spacing can fine-tune the height of our microstructures, which can be employed for a wide range of in vitro cell attachment and cell mechanic studies. Moreover, studies have shown PDMS MSAs as a combined platform for diffraction and cell adhesion study [20]. Thus, as our results demonstrated, DEL-MSAs can be used for studying cell behavior on a grating for real-time visualization of cellular activity.

### 3.5. Protein Patterning through Microcontact Printing

Another field that broadly employs MSAs is the surface pattering of small molecules and proteins, which are found in a wide range of applications such as biosensors [73], disease diagnostic tools like detection of COVID-19 [74,75], cell culturing [40,76,77,78], and other analytical experiments [79]. Explicitly, PDMS MSAs are widely used because of their low cost of ownership and inherent hydrophobicity, which provides a favored surface for protein adsorption [78]. Thus, we were motivated to test our PDMS DEL-MSAs in protein patterning, yet another biological application.

For this study, three shapes were chosen: Bullet and Pillar were chosen for microstructures height, a crucial attribute for microcontact printing. Petal was chosen for its unique geometry. After adsorption of PLL-FITC protein, the selected MSA construct was stamped on a glass substrate, with and without applying extra weight. We noted that the weight of the PDMS substrate itself was 0.7 g.

**Figure 7:**
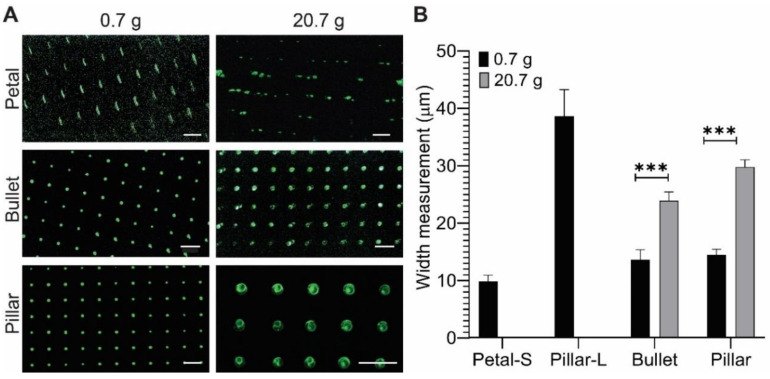
Microcontact Printing using MSAs. (**A**) Fluorescent microscopic images of the microcontact-printed PLL-FITC on a glass substrate. Two different weights were compared: 0.7 g (PDMS stamp only) and 20.7 g (plus 20 g weight). Petal did not hold the weight of 20.7g and yielded no proper transfer of protein. (**B**) Width of printed patterns. The additional weight significantly thickened the width of Pillar and Bullet printing by almost double. Error bars: STDEV. Statistical analysis: Student’s *t*-test (*** *p* < 0.001). (**A**) Scale bar: 100 μm.

In protein patterning under no extra weight condition (Figure 7A), the Petal substrate yielded an elongated line pattern (length −38 µm) with a bulging width (9 µm) along its axis. In contrast, Bullet and Pillar stamps yielded 13 µm and 14 µm diameter dots, respectively.

When a 20 g weight was applied on the stamps, Bullet and Pillar stamps generated 23 and 26 µm diameter dots, respectively, which were an approximately two-fold increase in the diameters compared to the no-weight condition (Figure 7B). However, the Petal MSAs collapsed, rendering no discernable protein pattern. With the application of weight on the substrate, the tips of the microstructures tend to collapse a little. Factors affecting the microstructure collapse are beyond the scope of this work, but they can be referred to in a previous study [2].

Furthermore, the overall blind area (where no protein was transferred) was lower than 5%, indicating the homogeneity and the high pattern fidelity of the protein transfer from our microstamps. However, when external pressure was applied, the defects increased to 8% in Bullet and 11% in Pillar, whereas the Petal substrate collapsed. These defects included faded fluorescent dots and dark spots within the patterned dots (Figure 7A). The increase in defects, with external pressure, can be attributed to various factors such as geometrical defects during fabrication, amount of protein transferred to the stamp, and contact pressure distribution. Nevertheless, these results demonstrably show the stability of DEL-MSA microstamps with and without external pressure.

Bullet and Pillar substrates rendered a protein pattern of radius as low as 6 µm. In general, to achieve protein patterning in the range of 1–10 µm, a chrome mask [76] and complex setup were used for master fabrication [80]. However, in our DEL technique, we have demonstrated a high-resolution patterning, in a range of a few microns, through a film photomask-based photolithography. Furthermore, the Petal substrate rendered an elongated line with nine microns axis-bulge and defined spacing, which could be used in the study of neuronal guidance. Together, these results proved that the structural attributes (wider base with hemispherical peaks) of the microstructure make the DEL-MSA microstamps applicable for protein micro-patterning.

## 4. Conclusions

Here, the DEL technique produced tapered MSAs with defined spatial aspects. The changes in exposure energy, photomask orientation, and wait time have produced varying aspect ratios, shape, and structural attributes of the MSA using SU-8 photoresist and PDMS soft lithography. Dimensions of the MSAs achieved in this study are as tall as 110 µm height and as short as 9 µm width, demonstrating the capability of this method for further exploration to create high-aspect-ratio MSA. Two of the substrates fabricated in this study have shown superhydrophobicity and Cassie–Baxter wetting state (very little wetting of the surface). Additionally, the optical properties of the MSA substrates were demonstrated using diffraction study. The variation of photomask rotation and lithography parameters has been shown to impact the diffraction pattern of the substrates directly. The biological application of the selected MSA has been validated through cell patterning, where the topographical effect of the tallest microstructures (Bullet and Pillar) was shown to influence the cell patterning on the substrates. Finally, microcontact printing rendered as small as ~6–7 µm dot patterns (Bullet and Pillar) and a line pattern of 9 µm width (Petal). Thus, the immediate applications of our device can shortly be extended to creating anti-wetting surfaces for studying cell mechanics, waveguided optics, cell adhesion, or cell patterning.

## Figures and Tables

**Table 1:** Fabrication parameters. Plain polydimethylsiloxane (PDMS), without any microstructure array (MSA), is defined as the control. Samples are named after the microstructure shapes.

Sample	Change in Parameter	First Exposure Energy (mJ/cm^2^)	Wait Time between the Exposures (s)	The Angle of Rotation (°)	Second Exposure (mJ/cm^2^)
Dome	Initial parameters	350	0	90	350
Petal	Half the angle of rotation	350	0	45	350
Bell	Half the exposures	175	0	90	175
Bullet	Wait time between exposures	350	60	90	350
Pillar	Half the exposures and wait time (From Bullet)	175	30	90	175

## Supplementary Materials

The following are available online at https://www.mdpi.com/2072-666X/11/10/903/s1, Figure S1: 3D view of the diffraction pattern from different DEL-MSA gratings. Right side: Red lines indicate the order of diffraction in the horizontal and vertical directions. Axis for all the figures is the same as indicated in the right-side Pillar image. The intensity distributions (indicated by z-axis) of all the sample shows that the intensity was high at the center and decreases with increasing order of diffraction. The bright intensity distribution also had different patterns for all the samples. Note that the brightest intensity observed on the right-side in the diffraction images was an artifact that occurred during image capturing and was not considered for any calculations or conclusions.

## References

[1] Gao J., Zhao J., Liu L., Xue W. (2016). Dimensional effects of polymer pillar arrays on hydrophobicity. Surf. Eng..

[2] Jin C., Qiao Q. (2016). Deformation of Pyramidal PDMS Stamps during Microcontact Printing. J. Appl. Mech..

[3] Chang Y.-J., Mohseni K., Bright V.M. (2007). Fabrication of tapered SU-8 structure and effect of sidewall angle for a variable focus microlens using EWOD. Sens. Actuators Phys..

[4] Lee J.-H., Choi W.-S., Lee K.-H., Yoon J.-B. (2008). A simple and effective fabrication method for various 3D microstructures: Backside 3D diffuser lithography. J. Micromech. Microeng..

[5] Evans C.J., Bryan J.B. (1999). “Structured”, “textured” or “engineered” surfaces. Cirp Ann..

[6] Liao X., Brown K.A., Schmucker A.L., Liu G., He S., Shim W., Mirkin C.A. (2013). Desktop nanofabrication with massively multiplexed beam pen lithography. Nat. Commun..

[7] Altuna A., Gabriel G., de la Prida L.M., Tijero M., Guimerá A., Berganzo J., Salido R., Villa R., Fernández L.J. (2010). SU-8-based microneedles for in vitro neural applications. J. Micromech. Microeng..

[8] Kuo J.-N., Hsieh C.-C., Yang S.-Y., Lee G.-B. (2007). An SU-8 microlens array fabricated by soft replica molding for cell counting applications. J. Micromech. Microeng..

[9] Li H., Chen F., Biria S., Hosein I.D. (2019). Prototyping of Superhydrophobic Surfaces from Structure-Tunable Micropillar Arrays Using Visible Light Photocuring. Adv. Eng. Mater..

[10] Shew B., Kuo C., Huang Y., Tsai Y. (2005). UV-LIGA interferometer biosensor based on the SU-8 optical waveguide. Sens. Actuators Phys..

[11] Zhao Y., Zhang X. (2006). Cellular mechanics study in cardiac myocytes using PDMS pillars array. Sens. Actuators Phys..

[12] Fu J., Wang Y.-K., Yang M.T., Desai R.A., Yu X., Liu Z., Chen C.S. (2010). Mechanical regulation of cell function with geometrically modulated elastomeric substrates. Nat. Methods.

[13] Hosokawa K., Hanada K., Maeda R. (2001). A polydimethylsiloxane (PDMS) deformable diffraction grating for monitoring of local pressure in microfluidic devices. J. Micromech. Microeng..

[14] Pishgar R., Wijdenes P., Iqbal F., Haidar K., Syeda A., Syed N., Dalton C. (2020). Impact of open surface area of multi-well microelectrode array on mammalian brain cells recording efficiency. Int. Soc. Opt. Photonics.

[15] Shimba K., Sakai K., Iida S., Kotani K., Jimbo Y. (2019). Long-term developmental process of the human cortex revealed in vitro by axon-targeted recording using a microtunnel-augmented microelectrode array. IEEE Trans. Biomed. Eng..

[16] Tsai Y.-C., Wu M.-D., Shih W.-P. (2011). A mask-free fabrication of SU-8/silicon spherical microprobe. Microelectron. Eng..

[17] Altuna A., de la Prida L.M., Bellistri E., Gabriel G., Guimerá A., Berganzo J., Villa R., Fernández L.J. (2012). SU-8 based microprobes with integrated planar electrodes for enhanced neural depth recording. Biosens. Bioelectron..

[18] Kim K., Lee J.-B. (2007). High aspect ratio tapered hollow metallic microneedle arrays with microfluidic interconnector. Microsyst. Technol..

[19] Rogers J.A., Jackman R.J., Schueller O.J., Whitesides G.M. (1996). Elastomeric diffraction gratings as photothermal detectors. Appl. Opt..

[20] Gibbons A., Láng O., Kojima Y., Ito M., Ono K., Tanaka K., Sivaniah E. (2017). Real-time visualization of cardiac cell beating behaviour on polymer diffraction gratings. RSC Adv..

[21] Kim S.-H., Lee S.-H., Kim Y.-K. (2002). A high-aspect-ratio comb actuator using UV-LIGA surface micromachining and (110) silicon bulk micromachining. J. Micromech. Microeng..

[22] Bustillo J.M., Howe R.T., Muller R.S. (1998). Surface micromachining for microelectromechanical systems. Proc. IEEE.

[23] Zhang X., Liu K., Chen K., Xu J., Ma Z., Li W., Xu L., Huang X. (2008). Fabrication and characterization of Si nanotip arrays for Si-based nano-devices. Int. Soc. Opt. Photonics.

[24] Tsai J.-C., Hsu Y.-S. (2011). Profile of microlens fabricated by the thermal reflow process. IEEE Trans. Magn..

[25] Jiang G., Baig S., Wang M.R. (2012). Prism-assisted inclined UV lithography for 3D microstructure fabrication. J. Micromech. Microeng..

[26] Taniguchi J., Koga K., Kogo Y., Miyamoto I. (2006). Rapid and three-dimensional nanoimprint template fabrication technology using focused ion beam lithography. Microelectron. Eng..

[27] Derby B. (2010). Inkjet printing of functional and structural materials: Fluid property requirements, feature stability, and resolution. Annu. Rev. Mater. Res..

[28] Grob L., Yamamoto H., Zips S., Rinklin P., Hirano-Iwata A., Wolfrum B. (2020). Printed 3D Electrode Arrays with Micrometer-Scale Lateral Resolution for Extracellular Recording of Action Potentials. Adv. Mater. Technol..

[29] Hu Y., Lao Z., Cumming B.P., Wu D., Li J., Liang H., Chu J., Huang W., Gu M. (2015). Laser printing hierarchical structures with the aid of controlled capillary-driven self-assembly. Proc. Natl. Acad. Sci. USA.

[30] Jafari R., Cloutier C., Allahdini A., Momen G. (2019). Recent progress and challenges with 3D printing of patterned hydrophobic and superhydrophobic surfaces. Int. J. Adv. Manuf. Technol..

[31] Cheng M., Deivanayagam R., Shahbazian-Yassar R. (2020). 3D printing of electrochemical energy storage devices: A review of printing techniques and electrode/electrolyte architectures. Batter. Supercaps.

[32] Kim K., Park D.S., Lu H.M., Che W., Kim K., Lee J.-B., Ahn C.H. (2004). A tapered hollow metallic microneedle array using backside exposure of SU-8. J. Micromech. Microeng..

[33] Ghosh S., Ananthasuresh G. (2016). Single-photon-multi-layer-interference lithography for high-aspect-ratio and three-dimensional SU-8 micro-/nanostructures. Sci. Rep..

[34] Hafeez H., Ryu H.-Y., An I.S., Oh H.-K., Ahn J.-H., Park J.-G. (2015). Dimensionally controlled complex 3D sub-micron pattern fabrication by single step dual diffuser lithography (DDL). Microelectron. Eng..

[35] Kwon K.Y., Weber A., Li W. (2014). Varying-length polymer microneedle arrays fabricated by droplet backside exposure. J. Microelectromech. Syst..

[36] SU-8 2000 Permanent Epoxy Negative Photoresist PROCESSING GUIDELINES FOR: SU-8 2100 and SU-8 2150, Kayakuam. https://kayakuam.com/wp-content/uploads/2019/09/SU-82000DataSheet2100and2150Ver5.pdf.

[37] Kang W.-J., Rabe E., Kopetz S., Neyer A. (2006). Novel exposure methods based on reflection and refraction effects in the field of SU-8 lithography. J. Micromech. Microeng..

[38] Xu F., Wu C.M., Rengarajan V., Finley T.D., Keles H.O., Sung Y., Li B., Gurkan U.A., Demirci U. (2011). Three-dimensional magnetic assembly of microscale hydrogels. Adv. Mater..

[39] Spaepen P., De Boodt S., Aerts J.-M., Vander Sloten J. (2011). Digital image processing of live/dead staining. Mammalian Cell Viability.

[40] Hart S.R., Huang Y., Fothergill T., Lumbard D.C., Dent E.W., Williams J.C. (2013). Adhesive micro-line periodicity determines guidance of axonal outgrowth. Lab. Chip.

[41] Hong C., Zhang X. (2018). Optically processed microlens array for single-beam lithography of plasmonic structures. Nanophotonics.

[42] De Boor J., Geyer N., Wittemann J.V., Gösele U., Schmidt V. (2010). Sub-100 nm silicon nanowires by laser interference lithography and metal-assisted etching. Nanotechnology.

[43] Quiñónez F., Menezes J., Cescato L., Rodriguez-Esquerre V., Hernandez-Figueroa H., Mansano R. (2006). Band gap of hexagonal 2D photonic crystals with elliptical holes recorded by interference lithography. Opt. Express.

[44] Walsh M.E. (2004). On the Design of Lithographic Interferometers and Their Application. Ph.D. thesis.

[45] Brueck S.R. (2005). Optical and interferometric lithography-nanotechnology enablers. Proc. IEEE.

[46] Crozier S.D. (2011). Development of Interference Lithography Capability Using a Helium Cadmium Ultraviolet Multimode Laser for the Fabrication of Sub-Micron-Structured Optical Materials.

[47] Chen X., Zaidi S.H., Brueck S., Devine D.J. (1996). Interferometric lithography of sub-micrometer sparse hole arrays for field-emission display applications. J. Vac. Sci. Technol. B Microelectron. Nanometer Struct. Process. Meas. Phenom..

[48] Bozler C.O., Harris C.T., Rabe S., Rathman D.D., Hollis M.A., Smith H.I. (1994). Arrays of gated field-emitter cones having 0.32 μm tip-to-tip spacing. J. Vac. Sci. Technol. B Microelectron. Nanometer Struct. Process. Meas. Phenom..

[49] Dendukuri D., Panda P., Haghgooie R., Kim J.M., Hatton T.A., Doyle P.S. (2008). Modeling of oxygen-inhibited free radical photopolymerization in a PDMS microfluidic device. Macromolecules.

[50] Alvankarian J., Majlis B.Y. (2015). Exploiting the oxygen inhibitory effect on UV curing in microfabrication: A modified lithography technique. PLoS ONE.

[51] Guerrero D.J., DiMenna W., Flaim T.D., Mercado R., Sun S. (2003). Dyed red, green, and blue photoresist for manufacture of high-resolution color filter arrays for image sensors. Int. Soc. Opt. Photonics.

[52] Varapnickas S., Malinauskas M. (2018). Processes of Direct Laser Writing 3D Nano-Lithography. Preprints.

[53] Zhang J., Chan-Park M.B., Conner S.R. (2004). Effect of exposure dose on the replication fidelity and profile of very high aspect ratio microchannels in SU-8. Lab. Chip.

[54] Li H., Fan Y., Conchouso D., Foulds I.G. (2015). Surface tension-induced PDMS micro-pillars with controllable tips and tilt angles. Microsyst. Technol..

[55] Sanjay S.L., Annaso B.G., Chavan S.M., Rajiv S.V. (2012). Recent progress in preparation of superhydrophobic surfaces: A review. J. Surf. Eng. Mater. Adv. Technol..

[56] Law K.-Y. (2014). Definitions for hydrophilicity, hydrophobicity, and superhydrophobicity: Getting the basics right. J. Phys. Chem. Lett..

[57] Miwa M., Nakajima A., Fujishima A., Hashimoto K., Watanabe T. (2000). Effects of the surface roughness on sliding angles of water droplets on superhydrophobic surfaces. Langmuir.

[58] Li X.-M., Reinhoudt D., Crego-Calama M. (2007). What do we need for a superhydrophobic surface? A review on the recent progress in the preparation of superhydrophobic surfaces. Chem. Soc. Rev..

[59] Papadopoulos P., Mammen L., Deng X., Vollmer D., Butt H.-J. (2013). How superhydrophobicity breaks down. Proc. Natl. Acad. Sci. USA.

[60] Cassie A. (1948). Contact angles. Discuss. Faraday Soc..

[61] Li F., Hou H., Yin J., Jiang X. (2018). Near-infrared light–responsive dynamic wrinkle patterns. Sci. Adv..

[62] Bajgrowicz-Cieslak M., Alqurashi Y., Elshereif M.I., Yetisen A.K., Hassan M.U., Butt H. (2017). Optical glucose sensors based on hexagonally-packed 2.5-dimensional photonic concavities imprinted in phenylboronic acid functionalized hydrogel films. RSC Adv..

[63] England G., Kolle M., Kim P., Khan M., Muñoz P., Mazur E., Aizenberg J. (2014). Bioinspired micrograting arrays mimicking the reverse color diffraction elements evolved by the butterfly Pierella luna. Proc. Natl. Acad. Sci. USA.

[64] Min K.-P., Kim J., Song K.D., Kim G.-W. (2019). A G-Fresnel Optical Device and Image Processing Based Miniature Spectrometer for Mechanoluminescence Sensor Applications. Sensors.

[65] Zhang J., Gai M., Ignatov A.V., Dyakov S.A., Wang J., Gippius N.A., Frueh J., Sukhorukov G.B. (2020). Stimuli-Responsive Microarray Films for Real-Time Sensing of Surrounding Media, Temperature, and Solution Properties via Diffraction Patterns. ACS Appl. Mater. Interfaces.

[66] Ouyang G., Wang K., Akram M., Henriksen L., Chen X. (2011). Optimization of PDMS network for a fast response and sensitive actuation material applied in a MEMS spatial light modulator. Appl. Phys. A.

[67] Ryabchun A., Wegener M., Gritsai Y., Sakhno O. (2016). Novel effective approach for the fabrication of PDMS-based elastic volume gratings. Adv. Opt. Mater..

[68] Suslik L., Pudis D., Goraus M., Nolte R., Kovac J., Durisova J., Gaso P., Hronec P., Schaaf P. (2017). Photonic crystal and photonic quasicrystal patterned in PDMS surfaces and their effect on LED radiation properties. Appl. Surf. Sci..

[69] Ghar A., Das U., Panigrahi P. Higher order micro transmission grating fabrication inside quartz glass by femtosecond laser micromachining. Proceedings of the 2017 IEEE Photonics Conference (IPC).

[70] Le Saux G., Magenau A., Böcking T., Gaus K., Gooding J.J. (2011). The relative importance of topography and RGD ligand density for endothelial cell adhesion. PLoS ONE.

[71] Kolind K., Dolatshahi-Pirouz A., Lovmand J., Pedersen F.S., Foss M., Besenbacher F. (2010). A combinatorial screening of human fibroblast responses on micro-structured surfaces. Biomaterials.

[72] Froeter P., Huang Y., Cangellaris O.V., Huang W., Dent E.W., Gillette M.U., Williams J.C., Li X. (2014). Toward intelligent synthetic neural circuits: Directing and accelerating neuron cell growth by self-rolled-up silicon nitride microtube array. ACS Nano.

[73] Mrksich M., Whitesides G.M. (1995). Patterning self-assembled monolayers using microcontact printing: A new technology for biosensors?. Trends Biotechnol..

[74] Yuan X., Yang C., He Q., Chen J., Yu D., Li J., Zhai S., Qin Z., Du K., Chu Z. (2020). Current and Perspective Diagnostic Techniques for COVID-19. ACS Infect. Dis..

[75] Hass K., Bao M., He Q., Park M., Qin P., Du K. (2020). Integrated Micropillar Polydimethylsiloxane Accurate CRISPR Detection (IMPACT) System for Rapid Viral DNA Sensing. bioRxiv.

[76] Oliva A.A., James C.D., Kingman C.E., Craighead H.G., Banker G.A. (2003). Patterning axonal guidance molecules using a novel strategy for microcontact printing. Neurochem. Res..

[77] Khadpekar A.J., Khan M., Sose A., Majumder A. (2019). Low Cost and Lithography-free stamp fabrication for Microcontact printing. Sci. Rep..

[78] Von Philipsborn A.C., Lang S., Bernard A., Loeschinger J., David C., Lehnert D., Bastmeyer M., Bonhoeffer F. (2006). Microcontact printing of axon guidance molecules for generation of graded patterns. Nat. Protoc..

[79] Bernard A., Renault J.P., Michel B., Bosshard H.R., Delamarche E. (2000). Microcontact printing of proteins. Adv. Mater..

[80] Schwaab D., Zentis P., Winter S., Meffert S., Offenhäusser A., Mayer D. (2013). Generation of protein nanogradients by microcontact printing. Jpn. J. Appl. Phys..

