# Supplementary material for: Fabrication of Tapered 3D Microstructure Arrays Using Dual-Exposure Lithography (DEL)
Source: Micromachines (Basel). 2020 Sep 29;11(10):903. doi: 10.3390/mi11100903 (PMC7599893; doi:10.3390/mi11100903)
Supplement: Supplementary file 1 [file micromachines-11-00903-s001.pdf]

micromachines

MDPI

Supplement Information

1. Apparent Contact Angles

Apparent contact angles were calculated and compared with the experimental value for Bullet and Pillar MSAs. Apparent contact angles were calculated from the Cassie-Baxter regimen as follows[60]:

$$\text{Cos } \theta c = \varphi(\text{Cos } \theta s + 1) - 1 \quad (1)$$

where θc and θs are the apparent water contact angles on the MSAs and a smooth surface, respectively, φ is the fractional interfacial area of the solid surface that is in contact with the water droplet. In other words, if the water droplet is sitting mostly on the air-pockets, φ approaches zero, leading to Cos θc approaching -1 and hence, θc approaches 180^0.

The fractional interfacial area was calculated from the measurements of the microstructures and the formula[9]:

$$\Phi = (\pi r^2 + 2\pi rh)/S^2 \quad (2)$$

where "r" is the radius of the tip of the microstructure, and "h" is the height of the wetting area below the tip, and "S" is the distance between the microstructures. Value from equation 2 was plugged into equation 1 to derive the apparent contact angle measurement for Bullet and Pillar MSAs.

Bullet and Pillar microstructures have sharper hemispherical peaks with a radius "r" of 6.5 μm and 7 μm, respectively (calculated from the SEM images). The height, h, was measured as the height of the microstructure below the droplet (Bullet h = 30 μm and Pillar h = 40 μm) (Figure 2d). "S" is the distance between the microstructure (100 μm for both Bullet and Pillar). From these values, the fractional interfacial areas can be calculated through equation 2 and the apparent angle from equation 1. The calculated apparent angles were 152^0 for Bullet and 148^0 Pillar MSAs. These values agreed with the observed values. Minor discrepancies between the calculated and measured contact angles could be associated with the accuracy of measuring the radius of the microstructure tips, the value of h, and the distance between the array structures.

2. Theoretical Diffraction Angle

For theoretical verification of the observed diffraction angle value, the theoretical diffraction angle was calculated from Bragg's law[62]:

$$2d\text{Sin}\theta_D = m\lambda \quad (1)$$

where d is the density of the microstructures on the grating, m is the diffraction order, λ is the wavelength of the incident light. Density, d, can be calculated from:

$$d=1/N \quad (2)$$

where N is the number of microstructures per meter of the MSAs. Value from equation 4 was used in equation 3 to find the angle of diffraction θ_D. From SEM (Figure 2A&C), the distance between microstructures was 100 μm. Hence, the value of N was calculated as 10^4 microstructures/m. From this value, the calculated angle of diffraction for our first order diffraction is 0.17^0 for all the structures. This value is in the range of the calculated value, which shows that our model obeys Bragg's law.

Supplement Figures:

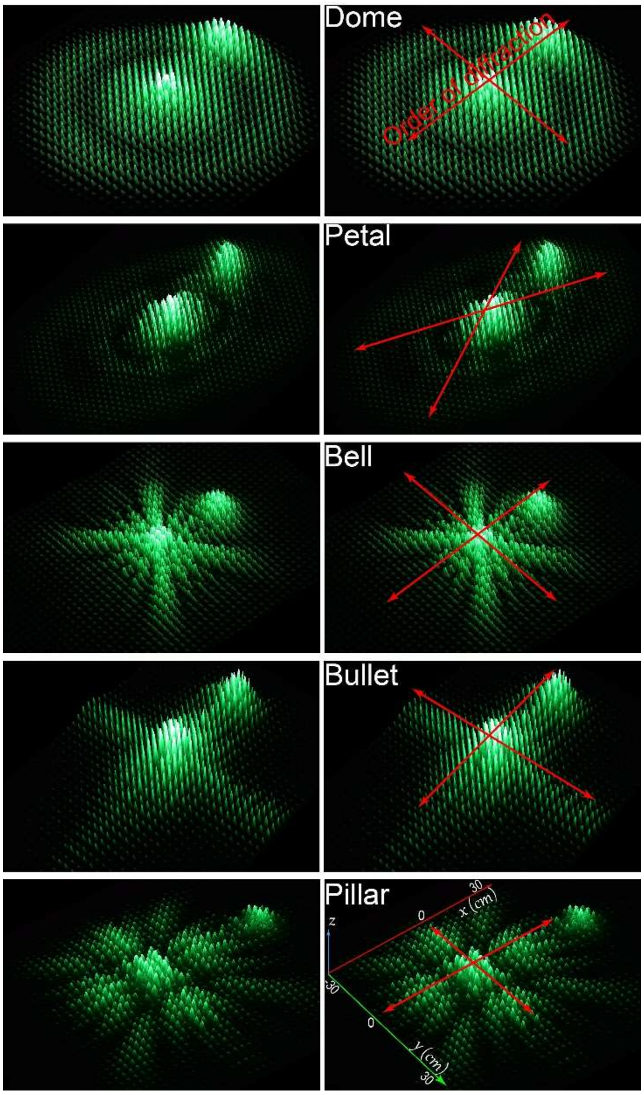

Figure S1. 3D view of the diffraction pattern from different DEL-MSA gratings. Right side: Red lines indicate the order of diffraction in the horizontal and vertical directions. Axis for all the figures is the same as indicated in the right-side Pillar image. The intensity distributions (indicated by z-axis) of all the sample shows that the intensity was high at the center and decreases with increasing order of diffraction. The bright intensity distribution also had different patterns for all the samples. Note that the brightest intensity observed on the right-side in the diffraction images was an artifact that occurred during image capturing and was not considered for any calculations or conclusions.
